# Regions with two amino acids in protein sequences: A step forward from homorepeats into the low complexity landscape

**DOI:** 10.1016/j.csbj.2022.09.011

**Published:** 2022-09-18

**Authors:** Pablo Mier, Miguel A. Andrade-Navarro

**Affiliations:** Institute of Organismic and Molecular Evolution, Johannes Gutenberg University Mainz, Hanns-Dieter-Hüsch-Weg 15, 55128 Mainz, Germany

**Keywords:** Protein sequence analysis, Low complexity regions, Linear motifs, polyXY

## Abstract

Low complexity regions (LCRs) differ in amino acid composition from the background provided by the corresponding proteomes. The simplest LCRs are homorepeats (or polyX), regions composed of mostly-one amino acid type. Extensive research has been done to characterize homorepeats, and their taxonomic, functional and structural features depend on the amino acid type and sequence context. From them, the next step towards the study of LCRs are the regions composed of two types of amino acids, which we call polyXY. We classify polyXY in three categories based on the arrangement of the two amino acid types ‘X’ and ‘Y’: direpeats (e.g. ‘XYXYXY’), joined (e.g. ‘XXXYYY’) and shuffled (e.g. ‘XYYXXY’). We developed a script to search for polyXY, and located them in a comprehensive set of 20,340 reference proteomes. These results are available in a dedicated web server called XYs, in which the user can also submit their own protein datasets to detect polyXY. We studied the distribution of polyXY types by amino acid pair XY and category, and show that polyXY in Eukaryota are mainly located within intrinsically disordered regions. Our study provides a first step towards the characterization of polyXY as protein motifs.

## Graphical abstract

**Figure:**
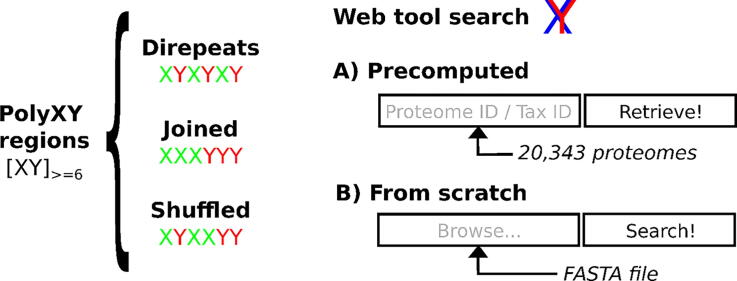

## 1 Introduction

Compositionally biased regions are frequent in protein sequences and are often not part of folded globular domains. Depending on the approach used to study them, these are sometimes identified as low complexity regions (LCR), and have been associated to disorder, aggregation, and structural flexibility [1]. Biased regions are found in proteins from all organisms and with various functions and subcellular locations [2]. The study of LCRs in protein sequences is gaining importance as it becomes increasingly evident that their distribution and conservation point towards their functionality [3], [4], [5]. However, the properties of these sequences, including flexibility and structures that depend on their molecular context, challenge technically and conceptually the approaches for the inference of relations between sequence, structure and function that have been traditionally used for the study of globular domains.

Consecutive stretches of a single repeated amino acid (polyX, or homorepeats) are the simplest LCRs [6]. Even though defining the minimal length of a functional polyX can be challenging (see e.g. for polyQ [7]), and experimental verification of the function and structure of such feature is scarce and time-consuming (e.g. [8], [9]), computational studies of homorepeats have provided us with a detailed picture of polyX extraordinary taxonomic distribution [10], [11], sequence context [2] and functions [12]. These studies show that particular polyX types in various taxonomic contexts are involved in protein interactions [13] and transcriptional regulation [14].

Inspired by the success of polyX computational studies, we reasoned that to facilitate a stepwise increase in our knowledge about LCRs, a next logical step should aim to characterize compositionally biased regions composed of two different amino acids (polyXY). The theoretical leap from studying polyX to polyXY regions is not trivial. For example, while polyX are periodic *per se*, the degree of periodicity of polyXY has to be evaluated and the possible effects discussed. Periodicity of LCRs is an important factor to consider, because while LCRs tend to be associated with a lack of structure, repetition can induce very stable structures. Homorepeats are already an example of this as polyQ can adopt beta, helical or disordered structure depending on neighbouring structures and interacting partners [7].

There are known cases of polyXY associated to biological functions; for example, RG-rich regions form intrinsically disordered regions found in numerous RNA-binding proteins and are involved in various physiological processes, e.g. transcription, splicing, DNA damage signaling and mRNA translation [15]. They also participate in liquid–liquid phase separation and formation of membraneless-organelles [16], and their expansion causes cellular toxicity and neurodegeneration [17]. Another example are the SR families of splicing factors present in metazoan organisms and plants, which combine an RNA binding domain (RRM) with a C-terminal RS-rich domain [18].

A previous approach studied protein regions consisting of two types of amino acids, but this study was limited to sequences from protein structures deposited in the Protein Data Bank (PDB) and was restricted to a small number of species [19]. Others have studied regions enriched in one, two or a few amino acids, limited to 4,227 protein families from five species [20]. No downstream analysis for polyXY regions was produced. Available methods to detect polyXY such as LCR-Composer [21] are not specific for regions composed of two different amino acids, and use a minimal length of 20 amino acids, which implies that short polyXY are not detected.

Here we study polyXY in all completely sequenced reference proteomes, a total of 20,340 proteomes, and classify these regions based on their composition and periodicity. We also provide insights into structural and functional features of polyXY with specific XY amino acid pairs.

## 2 Materials & methods

All complete reference proteomes available in the UniProtKB database release 2021_04 [22] were downloaded from their FTP site and classified per taxonomic group: 334 from Archaea, 8,134 from Bacteria, 1,805 from Eukaryota, and 10,067 from Viruses. Together they contain a total of 60,168,362 proteins. Additional taxonomic information was obtained for each species from the same site.

PolyXY were identified in the protein datasets using an in-house developed Perl script, which can be downloaded from https://cbdm-01.zdv.uni-mainz.de/∼munoz/polyxy/ for local use (accessed on 07 September 2022). No installation is needed. Dependencies are: Perl >= v5.28.0, and BioPerl libraries Bio:SeqIO and Getopt::Long. To identify polyXY, the script scans a protein sequence with a sliding window of 6 amino acids (minimum length). It then counts the occurrence of amino acids and considers the window as part of a polyXY if the number of different amino acid types found is two, and if both amino acids occur more than once. Then, the sliding window moves one position forward and the process is repeated. PolyXY are annotated by overlapping 6-residue windows that meet the conditions mentioned above.

Three categories of polyXY are considered: direpeats, joined and shuffled. Direpeats polyXY are regions covered in units ‘XY’ (or ‘YX’), allowing for one occurrence of X or Y alone, which can be at the termini of the region or between direpeats. For the purposes of polyXY classification, the order of the amino acids in the unit is irrelevant and they are given in alphabetical order. Joined polyXY are composed of a polyX followed by a polyY. For joined polyXY the order is relevant and it is recorded and reported. PolyXY not classified as direpeats or joined are classified as shuffled.

Positional annotations were downloaded from the UniProtKB database release 2021_04 [22], including domains and predicted disordered regions. For additional information regarding domain architectures and logo, we used the Pfam database v35.0 [23].

None of the proteins taken into account for the study of the structural conformation of the polyGL regions in bacteria had an experimentally solved structure. Thus, we relied on AlphaFold predictions with confidence threshold set as “Very high” [24]. Structures were represented with UCSF Chimera v1.15 [25].

Pairwise comparisons between distributions were tested for significance using non-parametric Mann–Whitney U statistical tests in R v3.6.3.

## 3 Results

### 3.1 The diversity of regions with two amino acids

**Fig. 1:**
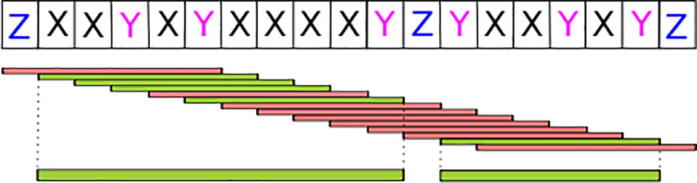
Detection of polyXY regions. A window of length six (bars; default parameter) slides over a protein sequence. At each position a test is run to check that only two types of residues are present and that each of them is detected at least twice. Green and red bars indicate positive and negative results of the test. The positives are overlapped to produce extended polyXY regions (thick green bars at the bottom). (For interpretation of the references to colour in this figure legend, the reader is referred to the web version of this article.)

We define polyXYs as regions with only two types of amino acids. To identify them in protein sequences, we use a sliding window of length six residues that detects polyXY if the residues found within are only ‘X’ or ‘Y’, and if each of them are present more than once. The resulting positive windows are joined together in extended polyXY regions if they overlap (see Methods for details and example in Fig. 1). The length of the window used gives us the minimal size of polyXY that can be reported, and was chosen to be six following previous works on homorepeats [2], [26].

Considering the polyXY detected, we highlight two special patterns: direpeats and joined. PolyXY direpeats are perfect short tandem repeats with unit ‘XY’ (or ‘YX’, order is not considered relevant; one adjacent half unit is accepted); these are characteristic of a few protein families (e.g. ‘SR’ repeats in the SR-family of splice factors mentioned above [18]). Joined polyXY corresponds to two consecutive homorepeats: polyX followed by polyY. A known example of the latter is polyQ followed by polyP [2]. It was observed that a polyP C-terminal from a polyQ decreases its propensity to aggregate, while it does not produce such an effect if situated N-terminally [8]. We believe there may be similar functional implications for other joined polyXY regions and therefore, the order of ‘X’ and ‘Y’ is relevant for this category.

We name all other polyXY not included in these two categories as shuffled, also encompassing those with a mixed nature (or imperfect). An example of this type of polyXY region is the polySG found in the human SMN protein (UniProtKB:Q16637), in positions 4–10 (‘SSGGSGG’). This polyXY is functional: the three serine residues in the region have been identified as being phosphorylated by protein kinase A, and described to be involved in the interaction of SMN with proteins Gemin2 and Gemin8 [27]. We will investigate if polyXY regions falling under each of the categories described above have distinct biological and structural features, also depending on the ‘X’ and ‘Y’ amino acid types.

### 3.2 Taxonomic distribution of polyXY regions

We scanned all completely sequenced reference proteomes from the UniProtKB database for polyXY regions, and found 32,236,246 polyXY regions in 18,898 different proteomes (20,340 proteomes were examined) (Table 1). All 1,442 proteomes lacking polyXY were viral, likely due to their low number of genes. Eukaryota presented a frequency higher than double of polyXY per protein compared to Archaea and Bacteria. A similar difference is found for other features typical of non-globular protein regions such as polyX [2], [28], internal repeats [29], LCRs [30] or intrinsically disordered regions [31]. Viruses have an even lower ratio of polyXY per protein.

**Table 1:** Datasets used in the analysis and general results.

	Viruses	Archaea	Bacteria	Eukaryota
Proteomes	10,067	334	8,134	1,805
Proteomes with polyXY	8,625	334	8,134	1,805
Proteins	514,486	760,947	30,426,472	28,466,457
Amino acids	122,184,712	212,010,960	9,737,445,508	12,457,985,469
PolyXY	114,309	226,401	10,290,833	21,604,703
Ratio PolyXY/Proteins	0.22	0.30	0.34	0.76
Ratio amino acids PolyXY / Proteins	0.0062	0.0069	0.0068	0.0118
Average polyXY length	6.63	6.48	6.45	6.78

Regarding the length of polyXY, average lengths are higher for Eukaryota. Viruses have slightly higher average length than Archaea and Bacteria, which could be because many viral proteins have been horizontally transferred from the eukaryotic host where they perform their function.

**Fig. 2:**
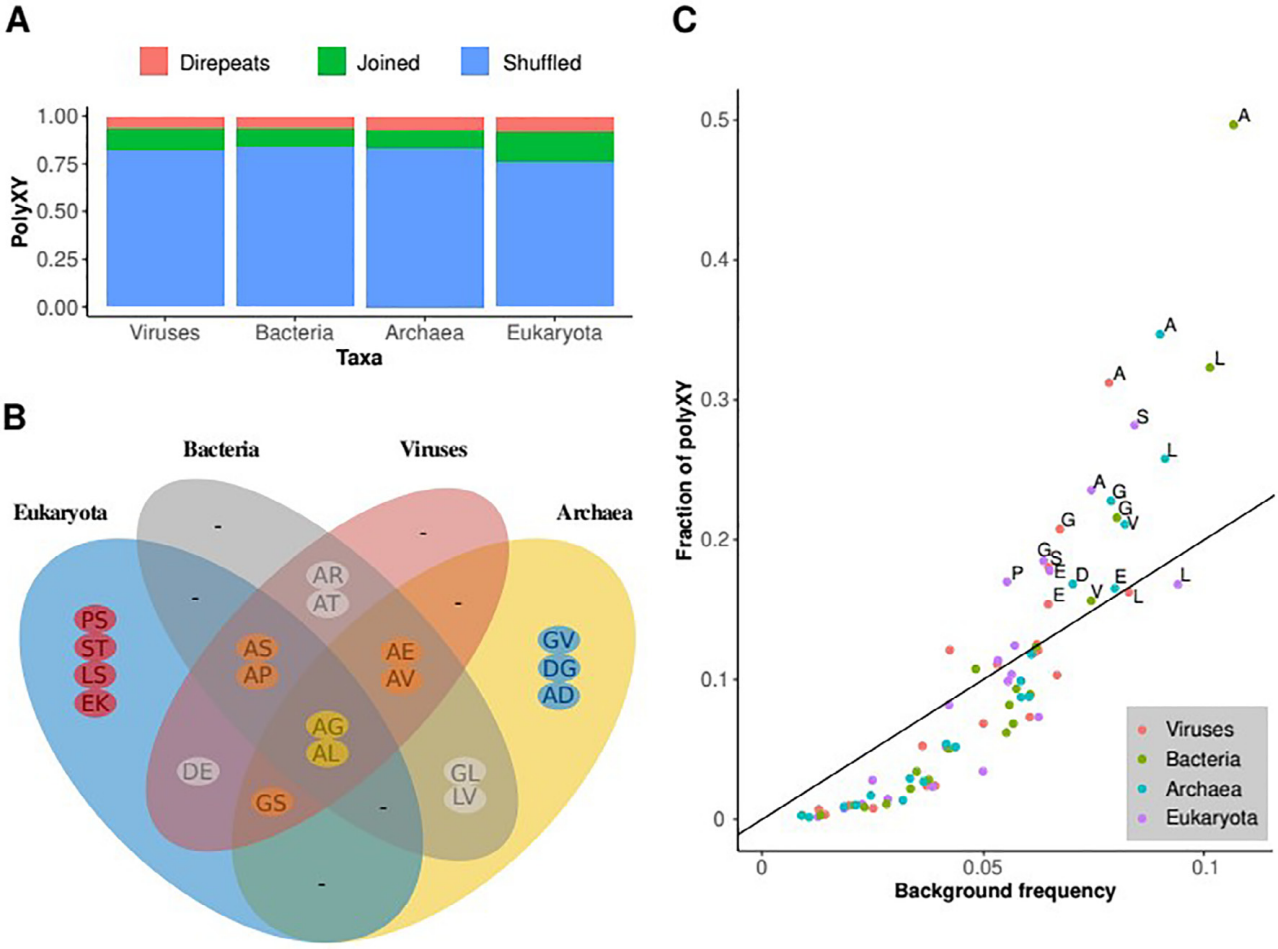
Description of polyXY features found in major taxonomic groups. (A) Proportion of polyXY per category detected in all the proteomes considered per taxa. (B) Top 10 most prevalent polyXY per taxa. (C) Fraction of polyXY regions containing a given amino acid per taxa, compared to the background frequency of the amino acid. Labels are shown for amino acids present in more than 15 % of polyXY regions. The x = y/2 line is indicated in black.

Regarding the polyXY categories, while the fraction of direpeats polyXY is rather constant across taxa, joined polyXY is more prevalent in Eukaryota than in the rest of the taxa (16 % in Eukaryota versus 9 %-11 % in the rest), corresponding to the higher abundance of polyX regions in Eukaryota [2], [28] (Fig. 2A).

A comparison of the top 10 most prevalent amino acid pairs forming the polyXY regions per taxa suggests high variability in the composition of the most used polyXY. Only polyAG and polyAL are highly used in the four taxa (Supplementary File 1; Fig. 2B). Regarding taxa-specific highly used polyXY, Viruses have none, which is expected given their strong functional and genetic dependency on their hosts, but it is surprising to find that Bacteria also have none.

Alanine is notable in that it is part of the two polyXY with top 10 (high) occurrence in the four taxa (‘AG’ and ‘AL’), and in four out of the five polyXY with high occurrence in three taxa. Alanine is one of the most frequent amino acids in all taxa but so is Leucine and this amino acid is rare in frequent polyXY (Fig. 2B; Supplementary File 2).

To see if there is a correlation between the frequency of amino acids and of polyXY types containing them, we compared these properties. We can observe that the most frequent amino acids occur in more polyXY regions (Fig. 2C; Supplementary File 2). The figure indicates the line x = y/2, to account for the fact that each polyXY is counted twice (once for amino acid ‘X’ and once for amino acid ‘Y’). The steeper slope of the distribution suggests that there is a tendency for frequent amino acids to cluster in polyXY regions. The plot shows the high prevalence of polyXY with alanine, which represents the highest fraction in three taxa (close to 50 % in bacteria), second to serine-containing polyXY in Eukaryota. Although leucine is similarly or more frequent than alanine, its use in polyXY is lower (Fig. 2C).

Next, we checked the relative position of the polyXY regions in the proteins containing them (Supplementary Fig. 1). We focused on the top 10 most prevalent polyXY per taxa, and considered the three categories separately. In general, the mean position of polyXY in proteins is the middle of the protein, suggesting that most polyXY have no positional preference. However, there are some types that deviate from this. There are cases in which polyXY regions from the three categories are positioned more towards the N- (‘AL’ and ‘LS’ in Eukaryota) or the C-terminal (‘DE’ in Viruses) part of the proteins. For some polyXY, two categories deviate from the middle towards the N- (‘GV’ in direpeats and joined of Archaea) or the C-terminal (‘AR’ in direpeats and joined of Viruses). And last, for some poly-XYone category deviate from the middle (‘AE’ in joined of Archaea, ‘AL’ in direpeats of Viruses, and ‘GL’ in direpeats of Bacteria).

### 3.3 Category-specific analysis

In previous sections, we have studied trends and biases of polyXY regions according to their composition. Here we will characterize polyXY according to the categories that consider the order of amino acids inside them: direpeats, joined and shuffled.

#### 3.3.1 Direpeats

**Fig. 3:**
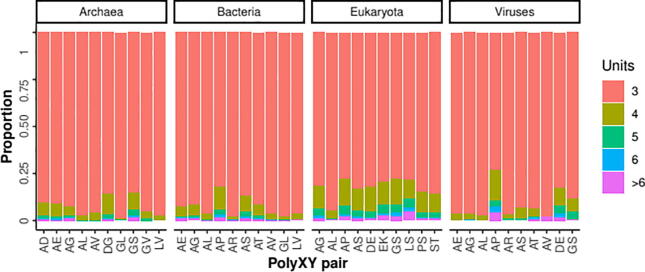
Proportion of direpeat polyXY regions per number of units. For the top 10 most abundant types per taxa.

Direpeats polyXY are characterized for the periodicity of the unit ‘XY’. Given our threshold of 6 residues for the detection of a polyXY, the minimum number of units observed is 3, but longer polyXY with 6 units and above are observed in all taxa considered (Table 2, Fig. 3). The proportion of direpeat polyXY with more than 3 units is higher in Eukarya than in the rest of the groups, in line with previous reports of low complexity regions being longer in eukaryotes [5].

**Table 2:** Number of repeats in direpeats polyXY.

All pairs	3 units	4 units	5 units	6 units	>6 units	Fraction >3 units
Eukaryota	1,400,308	231,915	71,595	29,371	44,802	0,21
Archaea	14,692	1,167	342	144	197	0.11
Bacteria	589,139	41,237	11,345	4,915	8,994	0.10
Viruses	6,562	561	178	86	160	0.13

**Fig. 4:**
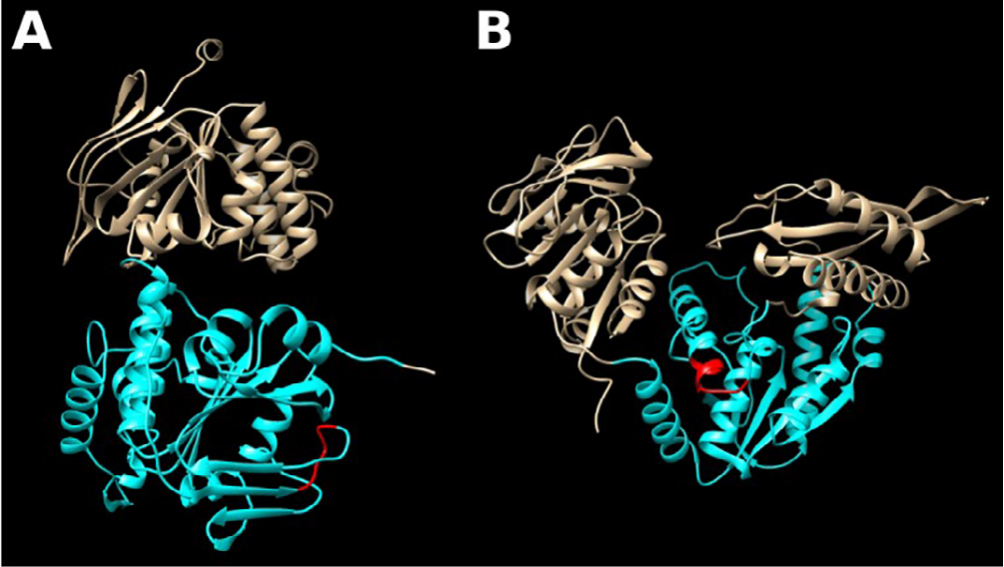
Structure models of direpeats polyGL regions. AlphaFold predictions for (A) Arabinose import protein AraG (UniProtKB:Q882I8; AlphaFold:AF-Q882I8-F1) and (B) GMP synthase GuaA (UniProtKB:Q5NG38; AlphaFold:AF-Q5NG38-F1), from bacteria Pseudomonas syringae pv. tomato and Francisella tularensis subsp. tularensis, respectively. Positionally annotated domains overlapping with the polyXY regions are colored in blue. The polyXY region, colored in red, is a direpeats polyGL. (For interpretation of the references to colour in this figure legend, the reader is referred to the web version of this article.)

As an example, we present the structural tendencies of direpeats polyGL regions in Bacteria. From the initial 33,728 direpeats polyGL in Bacteria, we took the 60 present in SwissProt proteins (58 unique proteins). We manually checked the overlap between the direpeats polyGL regions and their structural prediction from AlphaFold (Supplementary File 3). Out of the 39 regions with a very high prediction confidence, direpeats polyGL regions are 19 times part of an alpha helix, 14 times in coil, 5 in a helix and coil structure, and 1 within a beta strand. Only two of these regions overlap with a positionally annotated domain in UniProtKB, an ABC transporter 2 domain in the arabinose import protein AraG (Fig. 4A), and a GMPS ATP-PPase domain in the GMP synthase GuaA (Fig. 4B). Regarding the helix-coil cases, although they correspond to non-homologous proteins, in four out of five cases a last ‘LG’ unit follows a helix and adopts a coil C-terminal to the helix.

GMP synthase GuaA (glutamine amidotransferase) is an interesting example, because the protein is present in every organism as a component of the pathway for nucleotide de novo synthesis [32]. In a multiple sequence alignment of the family, the region around the direpeats polyGL in Francisella tularensis is conserved in length, that is, has no gap or insertion. While the region corresponding to the polyGL has a tendency to contain these G and L in other species (for example, the aligned human sequence is ‘LGRELGL’), in the multiple sequence alignment no single residue is fully conserved, while at the same time some surrounding positions are conserved. The results for this case suggest that a short direpeats polyGL could be capturing a structural motif with a propensity for alternating ‘GL’ units, like in this case a termination of a helix. Further studies will be needed to assess if such periodicity provides favourable structural properties.

#### 3.3.2 Joined

**Fig. 5:**
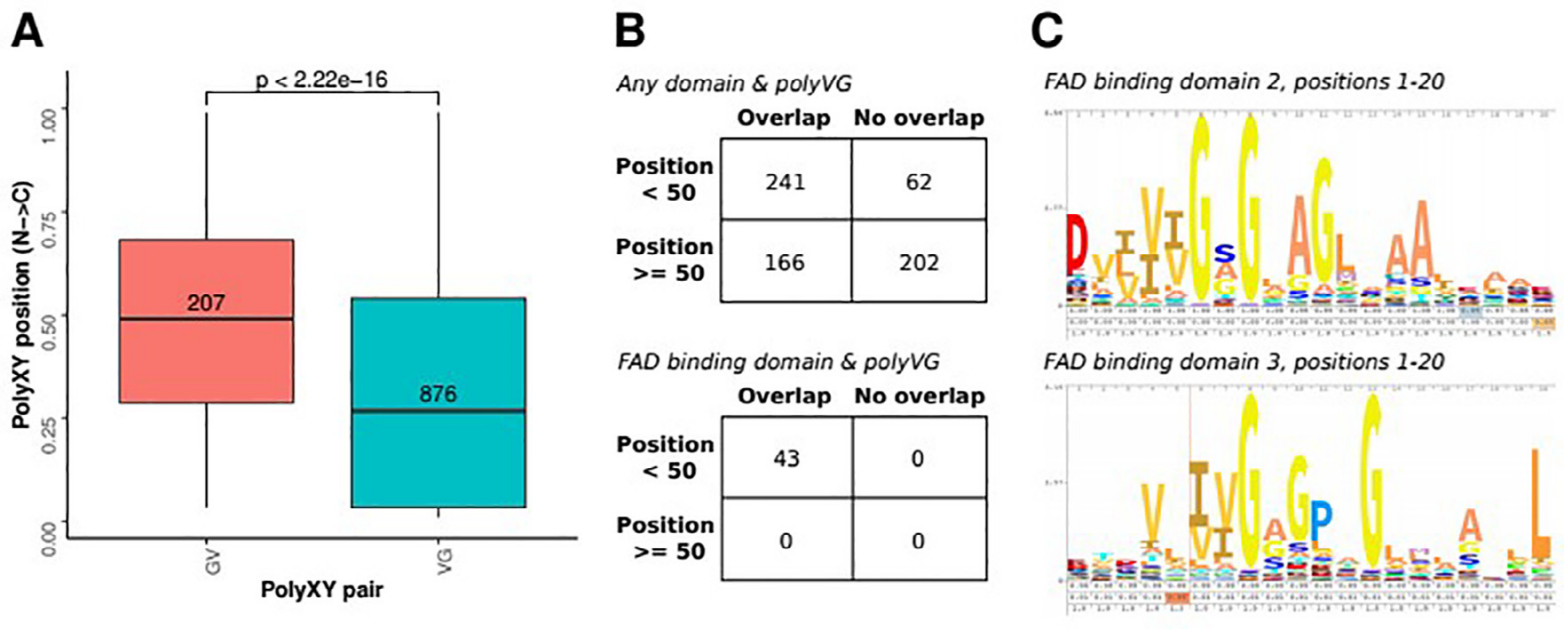
Joined polyVG prevails over joined polyGV in archaean proteins. (A) Relative position of joined polyGV and joined polyVG in archaean proteins; a non-parametric Mann–Whitney U statistical test was performed to compare the distributions. (B) Overlap of polyVG regions with any domain or with FAD binding domains, taken from the positionally annotated domains in the UniProtKB database, and considering the start position of the polyVG region before or after position 50 of the protein. (C) Positions 1–20 of the logos from the FAD binding domain 2 (PF00890) and FAD binding domain 3 (PF01494), obtained from the Pfam database.

Joined polyXY consist of a number of residues from one amino acid type followed by a number of residues from a different one. In this category we distinguish between joined polyXY and joined polyYX. For most XY pairs, the number of joined polyXY and joined polyYX are similar, but the numbers differ for some pairs (Supplementary Fig. 2). The most extreme result is for GV in Archaea, for which we find 207 joined polyGV and 876 joined polyVG. The joined polyVG in Archaea have a strong positional tendency towards the *N*-terminal of the protein (Fig. 5A).

We checked the overlap of the joined polyVG regions with any positionally annotated domain in the UniProtKB database, and found a clear overlap of polyVG regions with the FAD binding domain (Fig. 5B). This overlap is limited to the *N*-terminal part of the proteins. The FAD binding domain 2 (Pfam:PF00890) and FAD binding domain 3 (Pfam:PF01494) have an initial signature of several hydrophobic residues, and then several glycines (Fig. 5C). These domains have been annotated in more than 30,000 proteins, in which they can be found at the *N*-terminal part (∼60 %) or span over the whole protein (∼37 %); the polyVG region would then be always at the beginning of the protein. In viruses, which do not use this domain, the few cases found are almost equal for joined polyGV and polyVG (0.49 and 0.51, respectively; see taxonomy information for PF00890 in InterPro [33]). An example is the succinate dehydrogenase subunit A from *Halobacterium hubeiense,* with sequence ‘VVGGGG’ at positions 8 to 13. No structure is known for this protein but the AF model from a related sequence (AFDB:AF-P9WN91-F1 for UniProtKB:P9WN91; Fumarate reductase flavoprotein subunit from *Mycobacterium tuberculosis*) has ‘VVIGGGG’ from positions 8 to 14, with the valines and isoleucine forming the hydrophobic end of a beta strand which continues into a coil with the four glycines.

An example in a different domain but with known structure exists for the uridylate kinase of *Pyrococcus furiosus* (UniProtKB:Q8U122), for which the joined polyVG is ‘VVVGGG’. It occurs at positions 40 to 45 in domain PF00696 (Amino acid kinase family), it is present in all taxa except viruses, and it also has a tendency to be located in the *N*-terminal of proteins. Like in the previous example in the succinate dehydrogenase subunit A, here the three valines form the end of a beta strand that prolongs into a coil formed by the following three glycines. The signature of the domain displays a valine-rich region followed by a glycine-rich region in positions 38 to 44 of the profile.

These examples indicate that short joined polyXY can exist as part of globular functional domains and can be helpful to identify a common structural motif. These are manifested in a profile of the domain as a tendency for two consecutive amino acid rich regions. Further analyses would be required to identify if there are functional requirements for these particular residues in the context of the corresponding domains.

#### 3.3.3 Shuffled

The last category encompasses the polyXY regions that do not fall in any of the previous categories. This category is the most numerous, as it makes up to 75 %-80 % of all polyXY regions (Fig. 1A). We compiled the number of polyXY regions per amino acid pair and taxa from this category (Supplementary File 4).

**Fig. 6:**
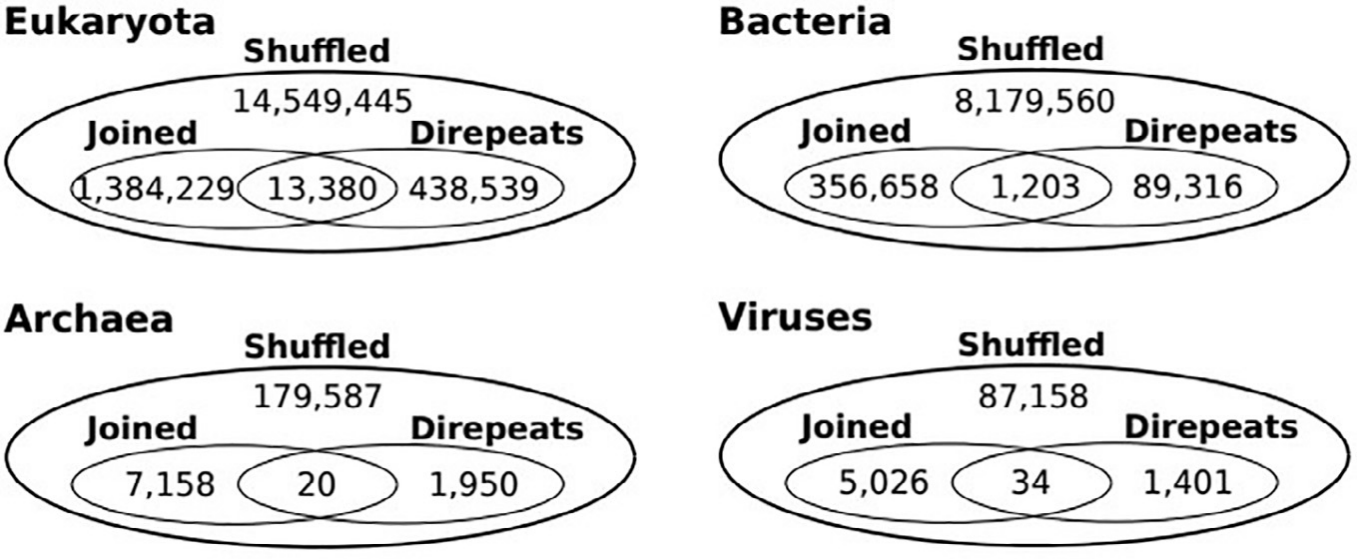
Categorization of shuffled polyXY. Number of shuffled polyXY regions per taxa containing a joined polyXY, a direpeat polyXY, or both.

We noticed that a number of smaller joined and direpeats polyXY are contained within longer polyXY reported as shuffled. We checked the frequency of this event and observed that the vast majority of the regions are purely shuffled, with a maximum of 8.45 % of them in Eukaryota containing a joined polyXY (versus 3.79 %-5.37 % in the rest of the taxa) (Fig. 6).

### 3.4 PolyXY in Eukaryota are mainly located within predicted intrinsically disordered regions

To assess if the polyXY cases discussed above where polyXY contributes to protein structure are the rule or the exception, here we study the overlap between the top 10 most abundant polyXY regions in proteins from Eukaryota to predicted intrinsically disordered regions (IDRs; defined by the MobiDB-lite method [34]) and to globular domains (defined by Pfam [23]). IDRs are parts of proteins that do not have a fixed 3D-structure, but instead can adopt multiple conformations under physiological conditions [35]. They are predicted from protein sequence analysis and are about three times more prevalent in eukaryotes than in prokaryotes [36]. IDRs contain a higher amount of low complexity sequences compared to their globular counterparts [37].

To compute that a polyXY overlaps a globular domain or an IDR, we require the complete polyXY to be within the other region. We considered for this analysis a total of 7,109,463 polyXY from 4,802,451 unique eukaryotic proteins with one or more polyXY. To have a baseline for comparison, for each individual polyXY in a given protein we also calculate the overlaps of a region of the same length in a random position of the same protein.

**Fig. 7:**
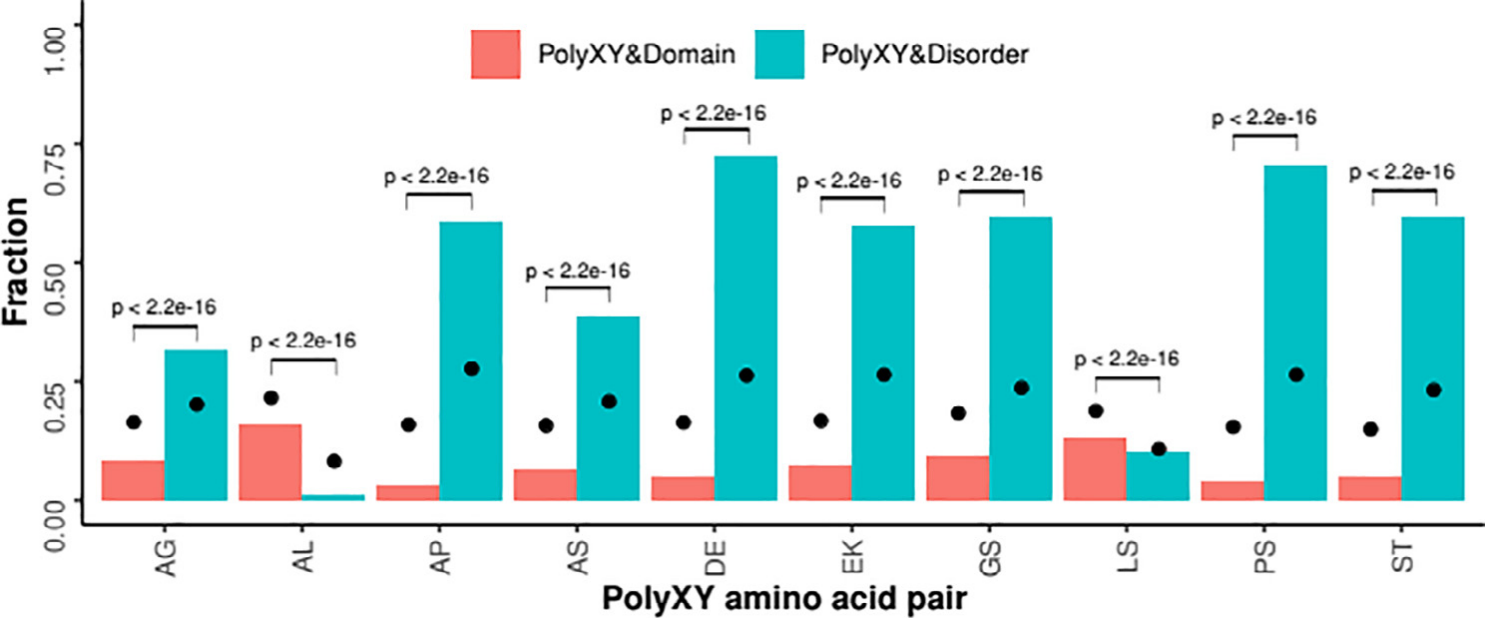
Overlap between polyXY, globular domains and disordered regions. Per polyXY region, a randomly-placed region with the same length was checked for overlap with a domain or a disordered region in the same protein (black circle). The top 10 most prevalent polyXY regions in Eukarya were considered. Non-parametric Mann–Whitney U statistical tests were performed to compare the distributions.

Results show that polyXY regions in eukaryotic proteins are less abundant in domains and more abundant in disorder than expected (Fig. 7). This is true for eight out of the ten most prevalent polyXY amino acid pairs. Exceptions are ‘LS’, for which there is little difference in the overlaps with both domains or disorder from the random results, and ‘AL’, which is enriched outside IDRs. IDRs generally lack hydrophobic amino acids, such as leucine, as they mediate in the co-operative folding that leads to long-range interaction and therefore to structure [35]. Results are not dependent on the category (direpeat, joined, shuffled) of the polyXY (Supplementary Fig. 3).

Together with our recently published work where we report that polyXY in IDRs have a higher tendency to overlap regions with experimental evidence for structure formation in comparison to the surrounding IDRs [38], these results suggest that polyXY could have a function in providing IDRs with the capability to adopt local structure.

### 3.5 The tool XYs to search polyXY regions from scratch

Our aim to characterize polyXY regions in this work is focused on complete proteomes. However, one may want to study these regions in a different dataset, or in a file containing proteins from various species. To that end, we have developed an easy-to-use web tool running the script and classification strategy described above. The XYs tool can be freely accessed here https://cbdm-01.zdv.uni-mainz.de/∼munoz/polyxy/, with no registration or installation needed (accessed on 07 September 2022). It has two working modes: A) precomputed or B) from scratch.

In mode A: precomputed, we have made available the results from the search of polyXY regions in the 20,340 complete reference proteomes we describe in this work. The user can access the results for a proteome by using the unique proteome identifier from UniProt, or the TaxID of a species. Default parameters were used for the search. We also provide the download of all 32,236,246 polyXY regions and their coordinates in one unique file. In mode B: from scratch, the user can search for polyXY regions in their own protein datasets. Additional options include the search of polyXY with: any X but a specific Y amino acid, pairs of specific X and Y amino acids, polyXY from one or more specific category, and only direpeats with a minimum number of repeated units. The user can also modify the minimum length of the polyXY region, and can merge close polyXY from the same amino acid pair separated by a maximum of one or two residues (other than amino acids X or Y).

Alternatively, we allow the user to download the source code and run the XYs script locally and without any installation (compatible with Unix/Linux systems). Only a protein FASTA file is needed in this case. Results include the ID of the protein containing the polyXY, start and end coordinates of the polyXY, amino acids forming it, its sequence, the polyXY category, and for direpeats polyXY, the number of repeated units.

As a case study, we computed the polyXY regions in the human proteome with both working modes, using the UniProtID ‘UP000005640′. Precomputed results are obtained right away. Details are shown for the 19,993 polyXY regions from the 20,588 proteins in the human reference proteome (UniProtKB release 2021_04), as well as a table with an overview of the number of polyXY regions per type (Supplementary File 5). On the other hand, to look for the regions from scratch, one can either upload a file with the proteome, paste the sequences in the text entry box, or use the proteome identifier ‘UP000005640′; in the latter option, the tool first downloads the proteome from the current UniProtKB release and then executes the search automatically. Execution time depends on the number of amino acids to be scanned; for example, for the proteome of *Saccharomyces cerevisiae* (‘UP000002311′, 6,060 proteins), the process takes 13 s. For the human proteome, the complete process takes 2 min. On September 2022, we obtained 46,402 polyXY regions from 79,740 proteins in the human proteome (not the reference one, thus the difference), using default parameters. Results are also divided by category: 3,384 direpeats, 6,486 joined, and 36,532 shuffled; these were calculated with the proteome downloaded on the day of the search, so updates in the database will result in slight changes of these numbers.

## 4 Conclusions

The study of polyXY regions is the logical continuation to the study of homorepeat regions in protein sequences as sequence motifs, considered as regions composed of two amino acid types instead of just of one.

We have defined, characterized and categorized the polyXY regions in 20,340 completely sequenced proteomes, considering two separate categories where the order of the ‘X’ and ‘Y’ residues matter: direpeats (XY alternate) or joined (a polyX is followed by a polyY); the reminder polyXY are defined as shuffled. The ratio between the number of polyXY regions and the proteins considered per taxonomic group is more than triple in Eukarya versus Viruses, and more than double versus Archaea and Bacteria. However, the relative amount of polyXY regions per category (direpeats, joined or shuffled) is similar between the taxa. PolyXY regions are both more abundant and longer in Eukarya, in line with previous reports for polyX regions [28].

PolyXY regions in eukaryotic proteins are depleted in domains and enriched in predicted disordered regions. We believe the tendency of polyXY regions to be out of domains has a structural reason. Low complexity regions usually differ in length even in closely related species, derived from a higher mutation rate [39]. They would be better accepted in regions with fewer structural constraints, thus they tend to be depleted in domains. Notwithstanding, the protein database we used to obtain the positional annotations of the domains may be not complete enough, and information about many domains could have been therefore not computed. However, that would not explain the fact that the overlap between the polyXY regions and globular domains is for most polyXY types almost half than for random regions of the same length as the polyXY.

Our work proposes a classification of polyXY regions, provides a method to identify them and suggests for the first time thresholds for polyXY identification. Overall, we did not observe great variation of properties of polyXY regarding function across taxa. It is possible that stricter thresholds will be necessary to differentiate functional polyXY; these will require taxa-specific approaches, maybe even at the level of individual protein families, and cannot be provided in the initial approach presented here. We believe that our work facilitates such future analyses.

## Funding

The authors received no specific funding for this work.

## Data Availability

The datasets generated in this work are available to download from the XYs web tool at http://cbdm-01.zdv.uni-mainz.de/∼munoz/polyxy/, with no restrictions for users.

## CRediT authorship contribution statement

**Pablo Mier:** Conceptualization, Methodology, Software, Validation, Formal analysis, Investigation, Resources, Data curation, Writing – original draft, Writing – review & editing. **Miguel A. Andrade-Navarro:** Conceptualization, Validation, Writing – review & editing, Supervision.

## Declaration of Competing Interest

The authors declare that they have no known competing financial interests or personal relationships that could have appeared to influence the work reported in this paper.

## Appendix A Supplementary data

Supplementary Fig. 1 Relative position of polyXY regions per category within the proteins (from 0 = N-terminal to 1 = C-terminal), for the top 10 most prevalent polyXY per taxa.

Supplementary Fig. 2 Ratio polyXY vs polyXY plus polyYX from joined polyXY. Top 10 most prevalent pairs per taxa are shown.

Supplementary Fig. 3 Overlap between polyXY, domains and disordered regions, divided by polyXY category. Per polyXY region, a randomly-placed region with the same length was checked for overlap with a domain or a disordered region in the same protein (black circle). The top 10 most prevalent polyXY regions in Eukarya were considered.

Supplementary File 1 Number of polyXY (Pair) occurrences per taxa (#PolyXY), also shown as percentage of proteins having them (%XY/Proteins), sorted by occurrence. Cell color indicates presence of the polyXY among the top 10 in each taxa, that is, region occupied by the polyXY in the Venn diagram of Figure 2B. Yellow, orange and gray indicate that they occur in multiple top 10 lists (all, three or two, respectively). Red and blue indicate that they occur in only one top 10 list (from Eukaryota or Archaea, respectively). There is no polyXY specifically occurring in the top 10 lists of Bacteria or Viruses.

Supplementary File 2 Frequencies of amino acids per taxa in proteins (Bg frequency) or as fraction of all polyXY found in the taxa with this amino acid (Fraction of polyXY). These values were represented in Figure 2C. Note that the sum of all values of background frequency for a taxa is 1.00, but the sum of all values of polyXY fraction is 2.00 because each polyXY is counted twice (once for amino acid X and once for amino acid Y).

Supplementary File 3 Structural predictions from AlphaFold for SwissProt bacterial proteins containing a direpeats polyGL region.

Supplementary File 4 Number of shuffled polyXY regions per XY pair and taxa. It is considered that polyXY = polyYX. The top 10 pairs per taxa are colored in red.

Supplementary File 5 Number of polyXY regions per XY pair in the human proteome. It is considered that polyXY = polyYX. The top 10 pairs are colored in red.
