# Supplementary figures and images for: Regions with two amino acids in protein sequences: A step forward from homorepeats into the low complexity landscape
Source: Comput Struct Biotechnol J. 2022 Sep 18;20:5516–23. doi: 10.1016/j.csbj.2022.09.011 (PMC9550522; doi:10.1016/j.csbj.2022.09.011)

## Archaea

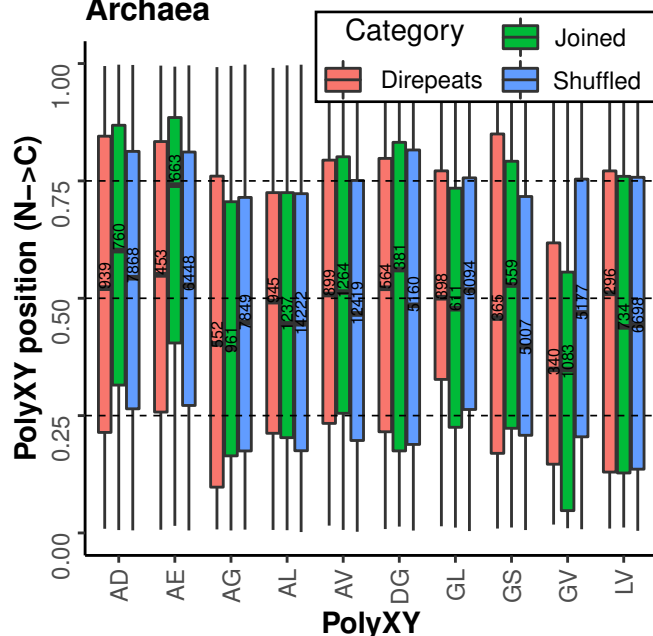

## Viruses

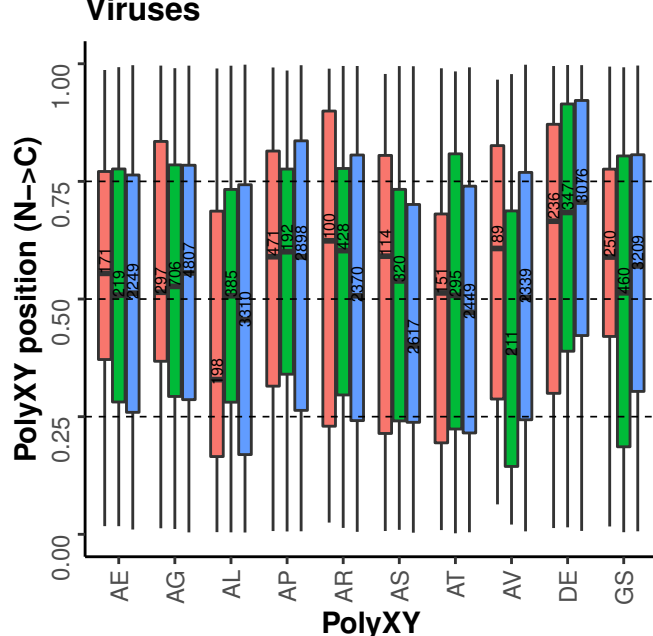

## Bacteria

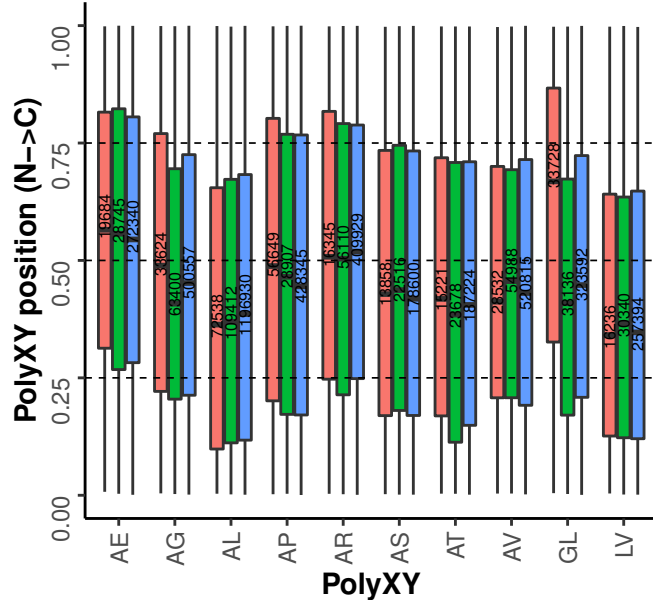

## Eukaryota

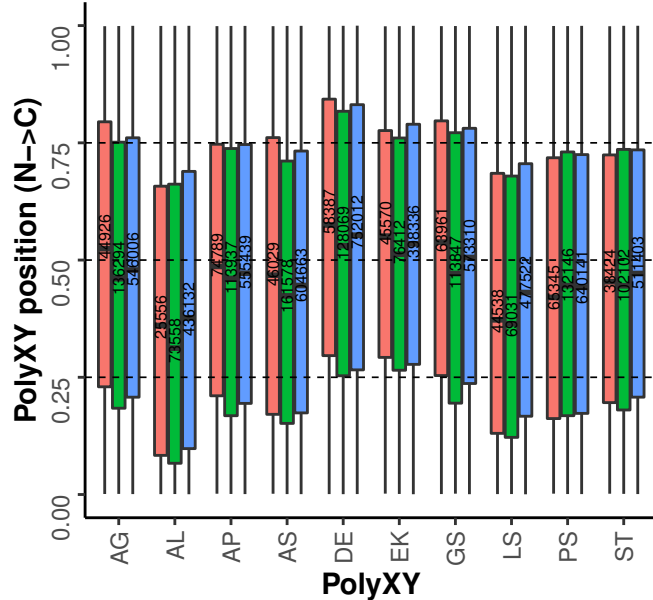

Supplement: Supplementary Fig. 1 — Relative position of polyXY regions per category within the proteins (from 0 = N-terminal to 1 = C-terminal), for the top 10 most prevalent polyXY per taxa. [file mmc1.pdf]

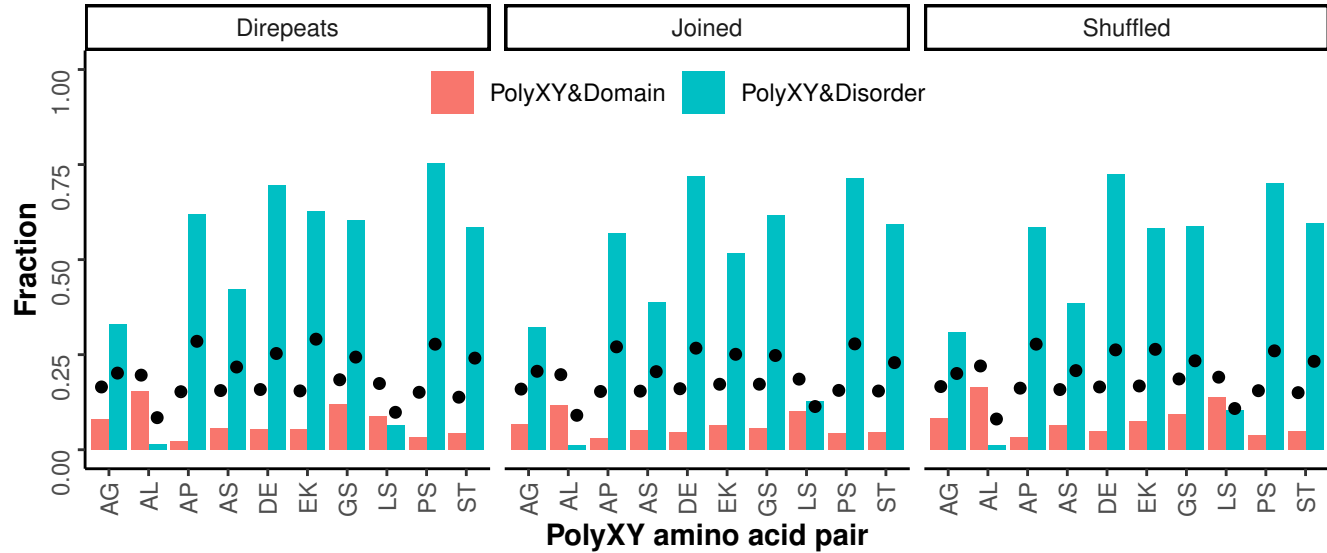

Supplement: Supplementary Fig. 3 — Overlap between polyXY, domains and disordered regions, divided by polyXY category. Per polyXY region, a randomly-placed region with the same length was checked for overlap with a domain or a disordered region in the same protein (black circle). The top 10 most prevalent polyXY regions in Eukarya were considered. [file mmc3.pdf]
