# Supplementary material for: Regions with two amino acids in protein sequences: A step forward from homorepeats into the low complexity landscape
Source: Comput Struct Biotechnol J. 2022 Sep 18;20:5516–23. doi: 10.1016/j.csbj.2022.09.011 (PMC9550522; doi:10.1016/j.csbj.2022.09.011)
Supplement: Supplementary Fig. 2 — Ratio polyXY vs polyXY plus polyYX from joined polyXY. Top 10 most prevalent pairs per taxa are shown. [file mmc2.pdf]

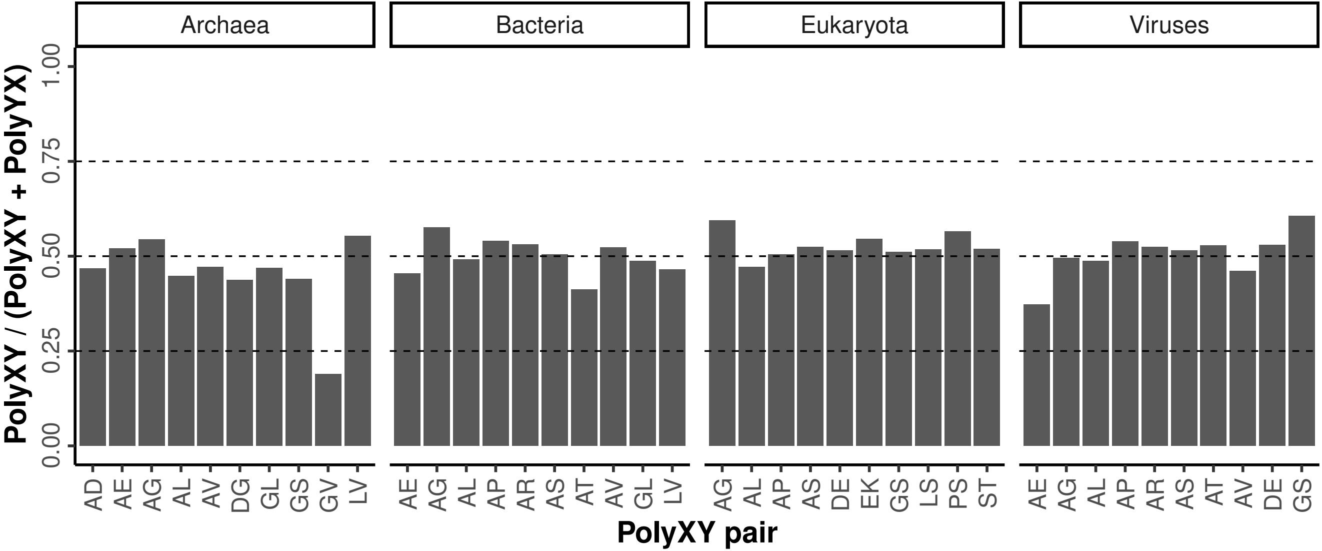

Archaea
Bacteria
Eukaryota
Viruses
PolyXY / (PolyXY + PolyYX)
1.00
0.75
0.50
0.25
0.00
AD AE AG AL AV DG GL GS GV LV
AE AG AL AP AR AS AT AV GL LV
AG AL AP AS DE EK GS LS PS ST
AE AG AL AP AR AS AT AV DE GS
PolyXY pair
